# Methods and applications of full-field optical coherence tomography: a review

**DOI:** 10.1117/1.JBO.27.5.050901

**Published:** 2022-05-20

**Authors:** Ling Wang, Rongzhen Fu, Chen Xu, Mingen Xu

**Affiliations:** a Hangzhou DianZi University, School of Automation, Hangzhou, China; b Key Laboratory of Medical Information and 3D Biological of Zhejiang Province, Hangzhou, China

**Keywords:** full-field optical coherence tomography, deep-learning enhanced FF-OCT, dynamic imaging, histological diagnosis

## Abstract

**Significance:**

Full-field optical coherence tomography (FF-OCT) enables *en face* views of scattering samples at a given depth with subcellular resolution, similar to biopsy without the need of sample slicing or other complex preparation. This noninvasive, high-resolution, three-dimensional (3D) imaging method has the potential to become a powerful tool in biomedical research, clinical applications, and other microscopic detection.

**Aim:**

Our review provides an overview of the disruptive innovations and key technologies to further improve FF-OCT performance, promoting FF-OCT technology in biomedical and other application scenarios.

**Approach:**

A comprehensive review of state-of-the-art accomplishments in OCT has been performed. Methods to improve performance of FF-OCT systems are reviewed, including advanced phase-shift approaches for imaging speed improvement, methods of denoising, artifact reduction, and aberration correction for imaging quality optimization, innovations for imaging flux expansion (field-of-view enlargement and imaging-depth-limit extension), new implementations for multimodality systems, and deep learning enhanced FF-OCT for information mining, etc. Finally, we summarize the application status and prospects of FF-OCT in the fields of biomedicine, materials science, security, and identification.

**Results:**

The most worth-expecting FF-OCT innovations include combining the technique of spatial modulation of optical field and computational optical imaging technology to obtain greater penetration depth, as well as exploiting endogenous contrast for functional imaging, e.g., dynamic FF-OCT, which enables noninvasive visualization of tissue dynamic properties or intracellular motility. Different dynamic imaging algorithms are compared using the same OCT data of the colorectal cancer organoid, which helps to understand the disadvantages and advantages of each. In addition, deep learning enhanced FF-OCT provides more valuable characteristic information, which is of great significance for auxiliary diagnosis and organoid detection.

**Conclusions:**

FF-OCT has not been completely exploited and has substantial growth potential. By elaborating the key technologies, performance optimization methods, and application status of FF-OCT, we expect to accelerate the development of FF-OCT in both academic and industry fields. This renewed perspective on FF-OCT may also serve as a road map for future development of invasive 3D super-resolution imaging techniques to solve the problems of microscopic visualization detection.

## 1 Introduction

From the perspective of resolution, radiation damage, price–performance ratio, and other aggregate indicators, optical imaging technology featuring high spatiotemporal resolution, high information capacity, and nonionizing properties have garnered significant research attention in the fields of biomedical and material science.^1^^,^^2^ Currently, high-resolution imaging technologies, such as fluorescence microscopy (FM),^3^^–^^5^ confocal microscopy (CM),^6^^,^^7^ two-photon microcopy (TPM),^8^^–^^11^ optical coherence tomography (OCT),^12^^,^^13^ and full-field optical coherence tomography (FF-OCT),^14^^,^^15^ have been widely adopted. Each technology has its distinct features. Combining fluorescence probe and optical microscopy, FM can perform micron-scale high-resolution imaging of tissues, organoids, cells, and other samples. However, FM cannot provide depth information and exhibits phototoxicity to tissues.^16^^,^^17^ Based on single-photon or two-photon excitation, CM and TPM render high imaging resolution and images with good contrast, but their imaging depth is mostly limited to a few hundred μm.^9^ Based on the principle of low coherent interference, OCT can provide histology-like images of a sample noninvasively; the transverse resolution and axial resolution of OCT can be controlled, respectively, which can reach 10  μm or even 1  μm; OCT imaging depth can reach 1 mm or higher, according to the scattering characteristics of samples, whereas the system cost is considerably lower than that of TPI. Therefore, OCT has attracted increased attention since it was first proposed in 1991.^12^^,^^13^^,^^18^

FF-OCT, which is developed from OCT, adopts a broadband light source with low spatial coherence to evenly irradiate the whole surface of the imaging sample and collects interferograms of different phases at the focusing depth of the sample by the planar detector, such as CMOS or CCD camera. Without performing transverse scanning, FF-OCT can reconstruct the optical slice image at the layer of the focal plane and the coherence plane as well as perform axial scanning similar to time-domain optical coherence tomography (TD-OCT) or time- and space-sharing parallel detection similar to Fourier-domain optical coherence tomography (FD-OCT).^14^^,^^19^ Therefore, optical slices with different focusing depths can be obtained to eventually reconstruct three-dimensional (3D) images of the sample. FF-OCT adopts lens with a high numerical aperture (NA) for sample imaging, which can significantly enhance its transverse resolution.^20^^,^^21^ The use of the broadband light source ensures high axial resolution, making the overall spatial imaging resolution of FF-OCT reach subcellular level, and the imaging results are comparable to biopsy.^15^^,^^22^ Based on full-field illumination, FF-OCT can conduct microscopy on two-dimensional (2D) slices at any depth within the imageable depth range of the sample without requiring transverse point scanning, thus correlating with conventional microscopy, CM, and TPM.^23^^–^^25^ In addition, FF-OCT can significantly suppress stray light using the coherent gate, given these advantages, FF-OCT has attracted extensive attention from researchers worldwide and demonstrated its applications in biomedicine, material detection, security identification, and other scenarios. As a type of optical microscopy, FF-OCT is advantageous in terms of resolution, penetration depth, imaging speed, noninvasiveness, and cost, but its application depends on the performance optimization of this technology, such as enhancing image reconstruction efficiency, optimizing image quality, extending imaging depth and field, and mining image information. In recent years, with the advancement of multimodal FF-OCT, particularly dynamic full-field optical coherence tomography (D-FFOCT), researchers can obtain the dynamic information of biological tissues and cells nondestructively, which further expands the application range of FF-OCT.^23^^,^^26^^–^^32^ It is of significance to review the research progress and application of FF-OCT to further promote the development of 3D high-resolution imaging technology and solve the problem of nondestructive detection in microscopic scale.

Beginning with an overview of the working principle and basic interference structure of FF-OCT, this work analyzes the possible optimization schemes of the system design based on the theoretical formula of its performance parameters. The latest research development of FF-OCT is reviewed in terms of imaging speed increase, imaging quality optimization, imaging flux enhancement, functional imaging extension, target information mining, and other performance improvements. The advantages and disadvantages of each specific imaging scheme are discussed along with its applicable scenarios. This work also provides examples of FF-OCT applications in biomedicine, materials science, security, and identification; further, it concludes by stating the technical bottlenecks in the development of FF-OCT.

## 2 Summary of FF-OCT Imaging

### 2.1 Working Principle and Basic Structure of FF-OCT

**Fig. 1:**
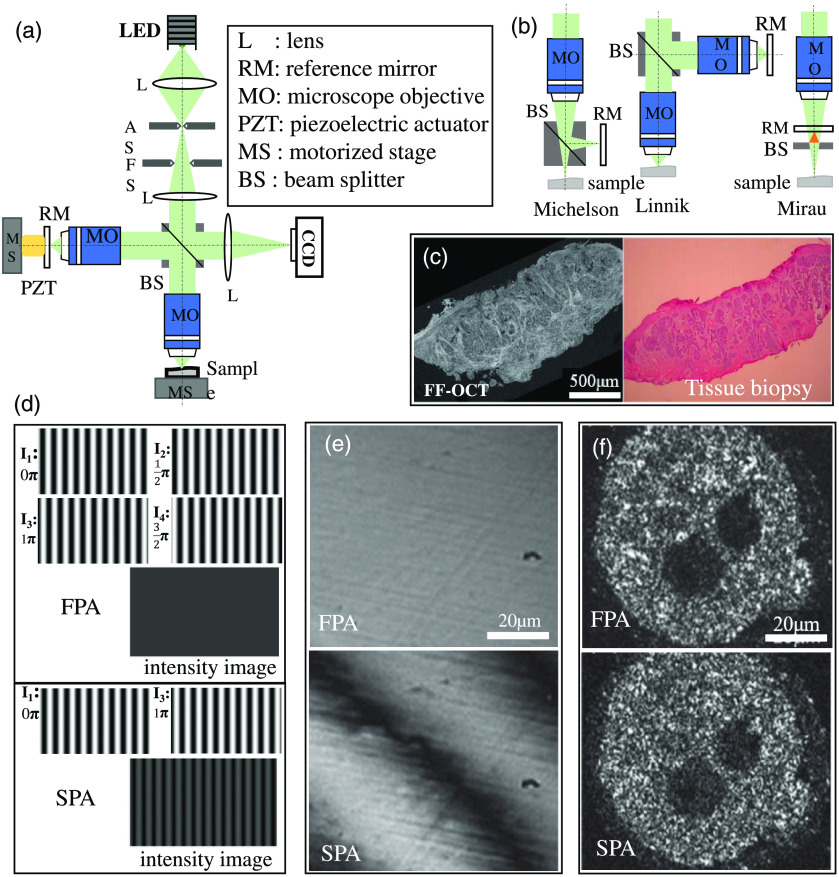
(a) Schematic diagram an FF-OCT. (b) Different optical set-ups of FF-OCT: Michelson-type, Linnik-type, and Mirau-type. Adapted from Refs. 19, 20, and 33. (c) FF-OCT images and tissue biopsy images of human forearm skin. Adapted from Ref. 22. (d) Tomographic reconstruction of simulated interference fringe with SPA and FPA, respectively. (e) Tomographic reconstruction of CaF2 crystal with SPA and FPA, respectively. (f) Tomographic reconstruction of embryo sample with SPA and FPA, respectively. Adapted from Ref. 19.

FF-OCT has simplified the system structure of conventional point scanning TD-OCT and line scanning FD-OCT by performing plane detection based on full-field illumination. Meanwhile, its acquisition speed is enhanced by full-field imaging via the CCD or CMOS camera.^14^ The imaging principle of the FF-OCT system is shown in Fig. 1. Its basic structure is a low coherence interference microscope system with a Linnik interference structure [Fig. 1(a)], spatial incoherent broadband light source. The plane array detector acquires the *en face* 2D image of the sample during one exposure.^20^ The frequently used interference structures in FF-OCT are shown in Fig. 1(b), including Michelson, Linnik, and Mirau interference structures.^14^^,^^20^^,^^33^^–^^35^ The Linnik interference structure is the most common type used in FF-OCT, whose two identical high NA microscope objectives are placed on the sample arm and the reference arm, respectively. The optical path of the two arms can be flexibly adjusted, and the optical path length as well as the focus can be modified independently. No other optical path exists between the objectives of the two arms and the imaging target, thereby extending the selectable range of NA and working distance of the objectives. Using microscope objectives with high NA, FF-OCT can achieve ultrahigh spatial resolution (axial and transverse) at the subcellular level and obtain real-time noninvasive images of the sample tissues [Fig. 1(c)], whose detection results are comparable to those of tissue biopsy. Besides the interference type, different overall structures of FF-OCT systems have been proposed, for example, external spectral modulation FF-OCT system, endoscopic FF-OCT system, and compact miniaturized FF-OCT system. In FF-OCT devices with external spectral modulation, imaging utilizing spectrally modulated, spatially incoherent illumination and a static Linnik interferometer. The first demonstration of spectrally modulated imaging through a multimode fiber optic bundle represents an important step toward achieving endoscopic FFOCM.^36^ In a compact miniaturized FF-OCT device, the interferometer part of the setup was designed to be as compact and light as possible ($11\times 11\times 5\;{\mathrm{cm}}^{3}$, 210 g), while keeping a Linnik interferometer design, which lays the foundations for a handheld device.^37^

The tomographic images of FF-OCT are reconstructed from the interference signal acquired by its detector. The reconstruction scheme varies based on different light sources and signal detection methods. According to the light sources and signal detection methods, FF-OCT is categorized into two types, i.e., time-domain full-field optical coherence tomography (TD-FFOCT, developed from TD-OCT) and Fourier-domain full-field optical coherence tomography (FD-FFOCT, developed from swept-source OCT).

TD-FFOCT adopts the spatial incoherent broadband light source for illumination and can obtain the tomographic interference spectrum data of the sample front without transverse scanning, thereby enabling rapid 3D imaging. In TD-FFOCT, the interference signal captured by plane array detector, contains sample information and serves as the basis of tomographic image reconstruction. The signal of each pixel in the interferogram can be expressed as

$$I=\bar{I}(x,y)+A(x,y)\cdot \cos[\phi (x,y)+\alpha ],$$ (1)

where $\bar{I}(x,y)$ is the DC signal, α is the single phase shift, ϕ(x,y) is the initial phase, and A(x,y) is the intensity signal containing the information of light reflectivity of the tissue at the focal depth. Equation (1) contains three unknowns, i.e., $\bar{I}(x,y)$, A(x,y), and ϕ(x,y). To analytically solve A(x,y), which is the intensity of the tomography, at least three interferograms with different phases are required. The results of obtained tomography would be better if more interferograms are involved during reconstruction.^19^ Nevertheless, the acquisition time and the computational load of interferograms would also increase accordingly. Commonly used reconstruction methods include three-step, four-step, and five-step phase-shifting algorithms as well as single-step phase-shifting algorithms (SPAs) based on the Hilbert transform.^38^^–^^43^

In general, to balance the acquisition time and reconstruction quality, the four-step phase-shifting algorithm (FPA) is the most adopted method for reconstruct tomography; the signals of four interferograms with a phase interval of π/2 are assigned as $I_{1}$, $I_{2}$, $I_{3}$, and $I_{4}$, corresponding to α as 0, π/2, π, and 3π/2, respectively, in Eq. (1). The intensity A(x,y) and phase ϕ(x,y) maps are calculated using Eqs. (2) and (3):

$$A(x,y)=\sqrt{{\left(I_{3}-I_{1}\right)}^{2}+{\left(I_{4}-I_{2}\right)}^{2}},$$ (2)

$$\phi (x,y)=\arctan(\frac{I_{4}-I_{2}}{I_{3}-I_{1}}).$$ (3)

The five-step method is similar to FPA, except that it performs reconstruction based on five interferograms with different phases; therefore, the reconstruction speed of the five-step method is slower than that of FPA; its intensity and phase maps can be calculated using Eqs. (4) and (5):

$$A(x,y)=\sqrt{4*(I_{2}-I_{4}{)}^{2}+(-I_{1}+2*I_{3}-I_{5}{)}^{2}},$$ (4)

$$\phi (x,y)=\arctan(\frac{2*(I_{2}-I_{4})}{2*I_{3}-I_{5}-I_{1}}).$$ (5)

The comparison of the imaging results of the FPA and SPA based on Hilbert transform is shown in Figs. 1(d)–1(f), in which tomographic reconstruction of simulated interference fringe and embryo sample was performed.^19^

Based on the Hilbert transform, SPA can reconstruct tomography with only two interferograms, thereby accelerating the imaging speed.^40^ The signals of interferograms $I_{a}$ and $I_{b}$ with a phase difference of θ are described using Eqs. (6) and (7):

$$I_{a}=\overset{¯}{I}(x,y)+A(x,y)\cdot \cos[\phi (x,y)],$$ (6)

$$I_{b}=\overset{¯}{I}(x,y)+A(x,y)\cdot \cos[\phi (x,y)-\theta ].$$ (7)

Here, θ is the phase shift, $\bar{I}(x,y)$ is the background signal, A(x,y) is the intensity signal required to be solved, and ϕ(x,y) is the initial phase signal. The analytical solution of intensity signal can be expressed by Eq. (8). The contrast of the tomography generated by this method is impacted by the value of θ, and the best contrast effect can be achieved when θ equals π; nonetheless, when θ reaches 0.7π, the contrast effect can already achieve 90% of the optimum:^42^

$$A(x,y)=\frac{1}{2\;\sin\;\frac{\theta }{2}}\cdot \sqrt{{\left(I_{a}-I_{b}\right)}^{2}+{\left[\text{Hilbert}(I_{a}-I_{b})\right]}^{2}}.$$ (8)

Notably, as shown in Figs. 1(d) and 1(e), the fringes cannot be completely eliminated in the reconstruction of FF-OCT tomographic interference images via SPA based on Hilbert transform. However, when SPA based on the Hilbert transform is adopted for the tomographic reconstruction of biological samples, almost no issue of residual fringes exists because the randomly distributed micro/nanostructures in biological samples make the fringes in the reconstructed image invisible, as shown in Fig. 1(f).^19^ A simplified algorithm for digital fringe analysis in two-wave interferometry with sinusoidal phase modulation was proposed in 2017. The algorithm consists of simple mathematical combinations of four frames obtained by integration by a camera of the time-varying intensity in an interference pattern during the four successive quarters of the modulation period. The algorithm is invariant by circular permutation of the four image frames. Any set of four consecutive frames can be used for the calculations, which simplifies the practical implementation of the method.^44^ In addition to the above-mentioned algorithms with sinusoidal phase modulation, there is also the “Larkin” algorithm for digital envelope detection, which is a simple but highly effective nonlinear algorithm for envelope detection in white light correlograms. The method is applicable to any bandpass signals where either the envelope or the phase, or both, need to be detected.^45^

**Fig. 2:**
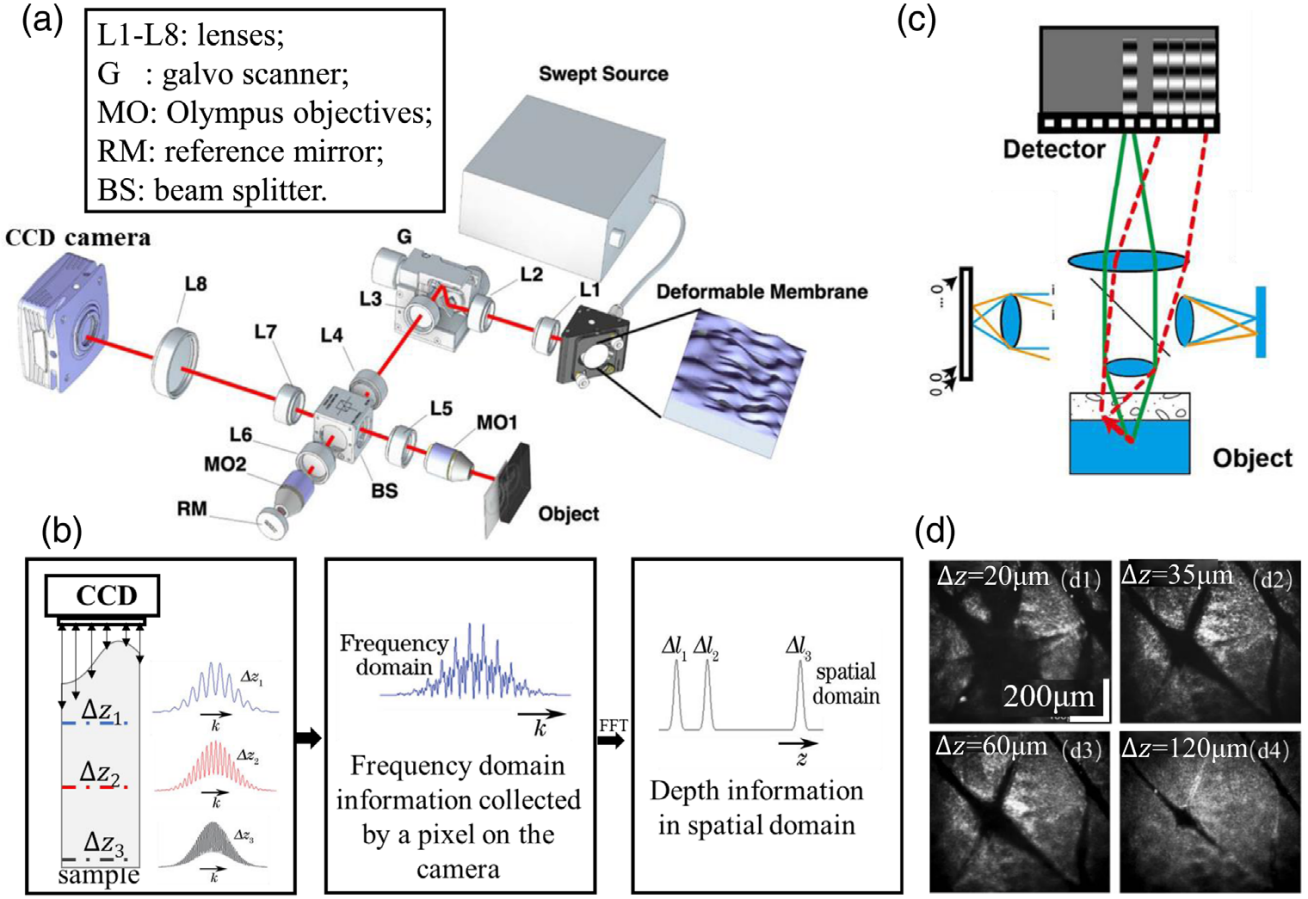
Schematic diagram of an FD-FFOCT system that can suppress crosstalk and speckle. (a) Optical set-up. (b) Reconstruction process of the FD-FFOCT system. (c) Crosstalk caused by incorrect backscattered ray mapping. (d) Human forearm skin images acquired by the cross-talk-free FD-FFOCT. (a) and (c) Reprinted from Ref. 46.

FD-FFOCT adopts spatial coherent swept light source combining planar array detector to perform time-sharing detection, which can achieve parallel detection in both frequency and spatial domain. The system structure is shown in Fig. 2(a); the axial information of spatial domain can be obtained from the frequency-domain information acquired by a pixel on the plane array detector.^46^^,^^47^ The image reconstruction aims to analyze the frequency-domain information of a plane (such as 512(x)×512(y)] then obtain the 3D volumetric data of spatial domain (as 512(x)×512(y)×1024(z)]; the processing flow is shown in Fig. 2(b). FD-FFOCT can conduct rapid 3D imaging without axial or transverse scanning, thereby overcoming motion artifacts caused by mechanical dither and repeated motion showing higher sensitivity and imaging speed. However, swept source is a type of spatial coherent light, and the backscattered light information of the corresponding sample points will be mapped to the wrong pixels during parallel detection, resulting in optical crosstalk and affecting the imaging results. The schematics of crosstalk generation are shown in Fig. 2(c). Reducing the spatial coherence of the light source or using multiangle illumination can diminish crosstalk and improve the imaging quality to a certain extent. Figure 2(d) shows the cross section of the 3D reconstruction of human forearm skin at different depths with reduced crosstalk.^46^

### 2.2 Key Performance Parameters of FF-OCT

The main performance parameters of conventional OCT and FF-OCT include axial resolution, transverse resolution, penetration depth, imaging field, imaging speed, detection sensitivity, and motion artifacts. Each performance parameter usually is mutually restricted.

The axial resolution of FF-OCT mainly depends on the coherence length of the light source, whose theoretical value is calculated using Eq. (9):

$$\delta_{z}=\frac{l_{c}}{2}\approx \frac{2\;\ln\;2}{n\pi }\cdot \frac{\lambda_{0}^{2}}{\Delta \lambda },$$ (9)

where $l_{c}$ is the coherence length, Δλ and $\lambda_{0}$ are the spectral bandwidth and central wavelength of the light source, respectively, and n is the refractive index of the medium, which equals 1 in air and is between 1.3 and 1.5 in biological tissue. Equation (9) can serve as a criterion for selecting light source.^48^

The transverse resolution of FF-OCT is determined by the half peak width of the transverse point spread function, whose theoretical value is calculated using Eq. (10):

$$\delta_{x}\approx \frac{\lambda_{0}}{2\mathrm{NA}}.$$ (10)

Here, NA is the numerical aperture of microscope objective; $\lambda_{0}$ is the central wavelength of the light source.

Imaging field, as a significant parameter of the system, is related to the NA of the microscope objective and the central wavelength of the light source; its theoretical value can be estimated using Eq. (11):

$$\mathrm{FOV}\approx \frac{0.3592\lambda_{0}}{NA^{2}}.$$ (11)

A large NA can increase the transverse resolution while reducing the imaging field. Therefore, it is inadvisable to excessively pursue high transverse resolution and neglect its adverse effects. In fact, FF-OCT can achieve subcellular level by just choosing an appropriate NA (0.3 to 0.5).

**Fig. 3:**
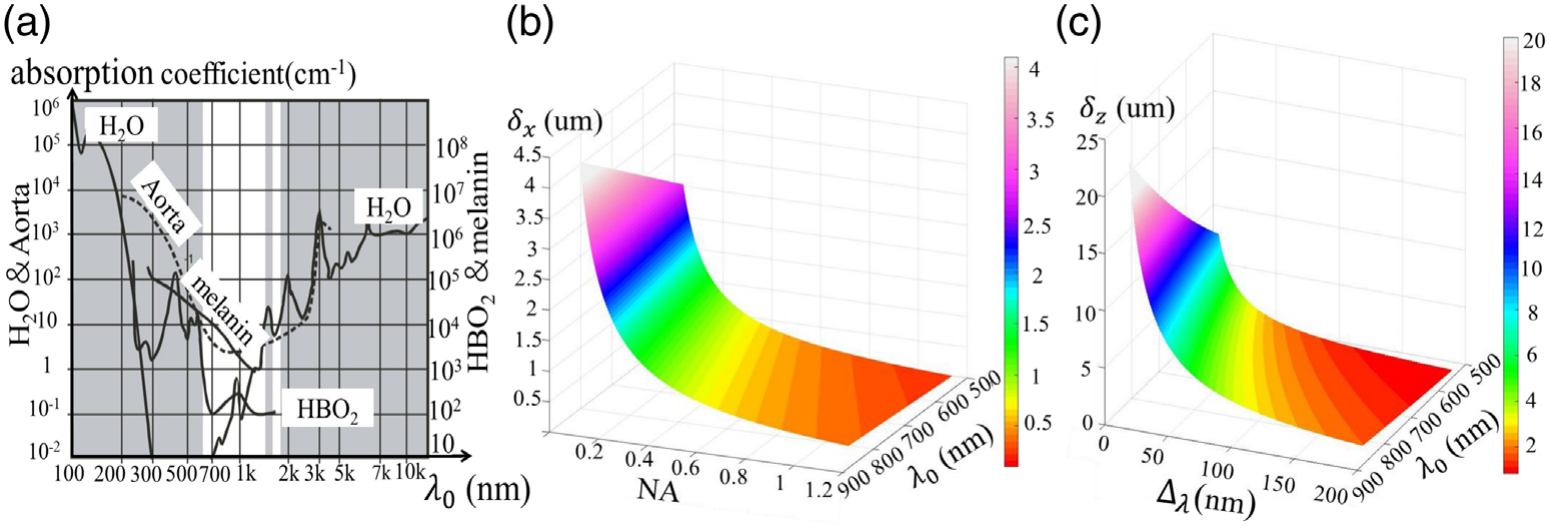
(a) Spectral absorption curve of common substances or molecules in biological tissues. Adapted from Ref. 48. (b) The influence of NA and center wavelength on transverse resolution. (c) The influence of spectral bandwidth and central wavelength on axial resolution.

Light source parameters, such as wavelength, bandwidth, spatial incoherence, and power will directly impact the imaging performance of the system. Figure 3(a) shows the absorption curves in each spectral band of several common molecules or substances in biological tissues, including water, aorta, oxygenated hemoglobin (HBO), and melanin. Considering the spectral response range and tissue imaging window of the plane array detector, the central wavelength of common light sources for FF-OCT is mainly 500 to 1000 nm.^44^ In this spectral band range and the NA range of 0.1 to 1.2, the transverse and axial resolutions of FF-OCT system are visualized, and the results are demonstrated in Figs. 3(b) and 3(c). The axial/transverse resolution data of typical light sources and microscope objectives are given here, as shown in Table 1. The types of light sources include light-emitting diode (LED), tungsten halogen lamp (THL), superluminescent diode (SLD), THL was first applied to the FF-OCT system in 2002.^50^ In the recent decade, due to the development of LED technology, the use of LED light sources became more and more common. These light sources are covered in Table 1. The central wavelength of the light source and NA of the microscope objectives can be preliminarily determined based on these results.

**Table 1:** Axial/transverse resolution data for common light sources and microscope objectives.

Parameters of the light source			Parameters of water immersion objectives		$\delta_{x}$	$\delta_{z}$
$\lambda_{0}$	Δλ	Type	NA	Magnification		
565 nm^49^	104 nm	LED	0.25 NA	10×	1.5 μm	1.35 μm
565 nm^41^	104 nm	LED	0.8 NA	10×	0.35 μm	1.35 μm
660 nm^31^	20 nm	LED	1.05 NA	30×	0.4	1
660 nm^27^	20 nm	LED	0.8 NA	40×	0.5 μm	1.7 μm
800 nm^50^	235 nm	THL	0.3 NA	10×	1.3 μm	1.2 μm
840 nm^14^	30 nm	LED	0.25NA	10×	2 μm	8 μm
850 nm^31^	40 nm	LED	0.3 NA	10×	1.7 μm	7.7 μm
1309 nm^39^	26 nm	SLD	*	*	70 μm	29 μm

Note: “*” indicates that no relevant data is provided in the paper.

The detection sensitivity of the system can be expressed by the minimum intensity signal that can be detected by the detector, which is one of the key parameters affecting the contrast and imaging depth of the tomogram. Its theoretical value can be calculated using Eq. (12):

$$R=K(\frac{\xi +\chi^{2}}{M*\xi })\frac{{\left(R_{\mathrm{inc}}+R_{r}\right)}^{2}}{R_{r}}.$$ (12)

The detection sensitivity is defined as 10×log(R), where K is a fixed constant, ξ is the full-well capacity of the camera, M is the superposition times of interference signals, χ is the electrical noise signal during data collection, $R_{\mathrm{inc}}$ is the light reflectivity of the sample, and $R_{r}$ is the light reflectivity of the reference mirror in the reference arm. The detection sensitivity of FF-OCT system can be enhanced by increasing the full-well capacity of the planar array detector, using a higher-power light source, and increasing the superposition times of interference signals.^27^ The performance of the camera affects that of the FF-OCT system; Professor W. Y. Oh’s team first applied an indium-gallium-arsenide (InGaAs) camera to the FF-OCT system in 2006. InGaAs area array cameras with a good responsivity in the 0.9 to 1.7  μm wavelength range may enable imaging at longer wavelengths, thereby increasing the penetration depth of FF-OCM.^51^ Moreover, the use of water or oil immersion on microscope objectives not only can improve the matching degree of the microscope objective and sample refractive index but also enhances the system sensitivity to a certain extent.^19^^,^^41^

## 3 FF-OCT Performance Optimization Methods

Compared with conventional OCT, the overall performance of FF-OCT is superior in terms of faster imaging speed and better resolution. However, in the practical application events of FF-OCT, constraints of the system implementation and insufficiency of image information extraction cannot be neglected. The overall performance and applicable scope of FF-OCT can be enhanced via technological innovation. The ways to improve performance can be divided into five directions: (1) quickening the imaging speed, (2) optimizing the imaging quality by improving the contrast and decreasing the dispersion, aberration, and motion artifacts, (3) extending the imaging depth and field of view (FOV), (4) additional contrast via multimodal imaging for providing complementary information in addition to structure; and (5) combining deep learning for image information mining. Optimization of the specific implementation scheme of FF-OCT can improve its application effect in the field of microscopic detection, thus enabling different application scenarios and requirements. The review of FF-OCT performance optimization methods can help researchers to fully understand this submicron high-resolution imaging technology and overcome the issues in system implementation under the requirements of innovative applications.

### 3.1 Increasing the Imaging Speed

The area array sensor and high NA microscope objectives adopted by FF-OCT can achieve submicron-level resolution while ensuring fast imaging speed. However, realizing real-time *in vivo* imaging remains challenging, and enhancing the overall imaging speed of the system is still a research focus. Based on the imaging principle of FF-OCT, reconstruction algorithms involving fewer interferograms as well as phase shifts with higher speed and accuracy can enhance the imaging speed of the system. The specific reconstruction algorithm of FF-OCT tomography is detailed in Sec. 2.1. The acquisition of interferograms with different phases is achieved by introducing a phase shift, which can be categorized into two methods depending on whether mechanical displacement is involved: mechanical phase shifting using the piezoelectric ceramic (PZT) phase shifters and instantaneous phase shifting without mechanical displacement using wave plates.

**Fig. 4:**
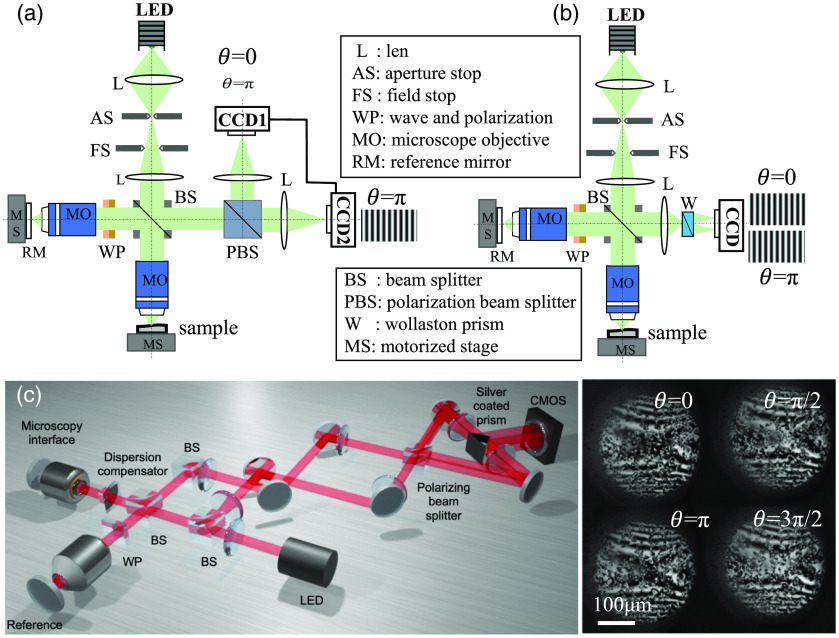
(a) Schematic diagram of dual-channel instantaneous phase-shifting FF-OCT set-up. Adapted from Ref. 40. (b) Instantaneous phase-shifting FF-OCT set-up of a single channel with two divisions. Adapted from Ref. 39. (c) Instantaneous phase-shifting FF-OCT set-up of a single channel quarter, and four interferograms collected at the same time with a phase interval of π/2. Scale bar: 100  μm. Reprinted from Ref. 41.

PZT phase shifting is the most common approach, which uses the piezoelectric effect to produce accurate small displacement to change the position of the reference mirror for phase shifting. PZT phase shifting has two methods, i.e., fixed-step phase shifting and carrier frequency phase shifting. The phase of the fixed-step approach changes discretely between intensity measurements, and the data acquisition is conducted after phase shifting. The carrier frequency method uses sine waves to enable the reciprocating motion of PZT and uses a square wave with a frequency four or five times the sine wave to trigger the detector and collect interferograms, which can accelerate the imaging speed but remains in the category of discrete phase shift.^50^ By combining the PZT phase shift with the instantaneous phase shift using wave plate, two movements of PZT can achieve four-step phase shift results by constructing the optical Hilbert transform.^52^ This dual-channel structure with two detectors can enhance the overall phase shifting speed. In the genuine instantaneous phase shift without mechanical displacement, the combination of a half-wave plate and linear polarizer introduces a phase difference of π between the optical paths of two arms; two interferograms with a phase difference of π are simultaneously acquired by two cameras. The SPA based on the Hilbert transform is then used to reconstruct the tomography, the construction of which is shown in Fig. 4(a).

Industrial cameras with high well capacity and high frame rate are expensive; thus, using two cameras would increase the system cost. The dichotomy of a single channel can be achieved using a Wollaston prism, then different sensor regions of one camera can simultaneously detect two quadrature polarized light fields, as shown in Fig. 4(b).^39^^,^^43^ Currently, the instantaneous phase-shifting structure with a quartered single channel has also been achieved, as shown in Fig. 4(c), which can simultaneously acquire four phase-shifting images using a single camera. Compared with the PZT four-step phase shift, this method can significantly enhance the imaging speed. Instantaneous phase-shifting approaches with a dichotomized or quartered single channel can reduce the system cost and enhance the imaging speed, but it would also decrease the number of pixels available for single imaging on an industrial camera, hence rendering a certain impact on the transverse imaging range of the system.

Each phase shifting approach varies in phase shifting speed and quality, economic cost, system complexity, and imaging field. Several phase shifting implementation schemes are listed in Table 2, which can serve as the reference for selecting phase-shifting approach during system design. The number of asterisks in Table 1 represents the degree of excellence regarding the performance, and more asterisks indicate a better performance.

**Table 2:** Features of different FF-OCT structures.

Reconstruction algorithm + the way of phase shifting	A channel divided area	Reconstruction speed	Reconstruction quality	CCD	Structure complexity
Single-step algorithm + PZT^42^	1	(***)	(*)	1	(*)
Three-step algorithm + PZT^19^	1	(***)	(*)	1	(*)
Four-step algorithm + PZT^14^	1	(**)	(***)	1	(*)
Five-step algorithm + PZT^19^	1	(*)	(***)	1	(*)
Single-step algorithm + wave plate + Wollaston prism^39^^,^^40^	2	(****)	(**)	1	(**)
Single-step algorithm + wave plate^52^	2	(****)	(**)	2	(**)
Four-step algorithm + wave plate + PZT^43^	2	(***)	(***)	2	(**)
Four-step algorithm + wave plate^38^^,^^41^	4	(****)	(****)	1	(***)

### 3.2 Enhancing Imaging Quality

To enhance imaging quality, the following aspects can be considered: reducing external noise to improve the accuracy and sensitivity of signal acquisition, reducing aberration to improve resolution, and reducing image noise to increase contrast. The FF-OCT image quality can be enhanced by either hardware or by algorithm and software.

**Fig. 5:**
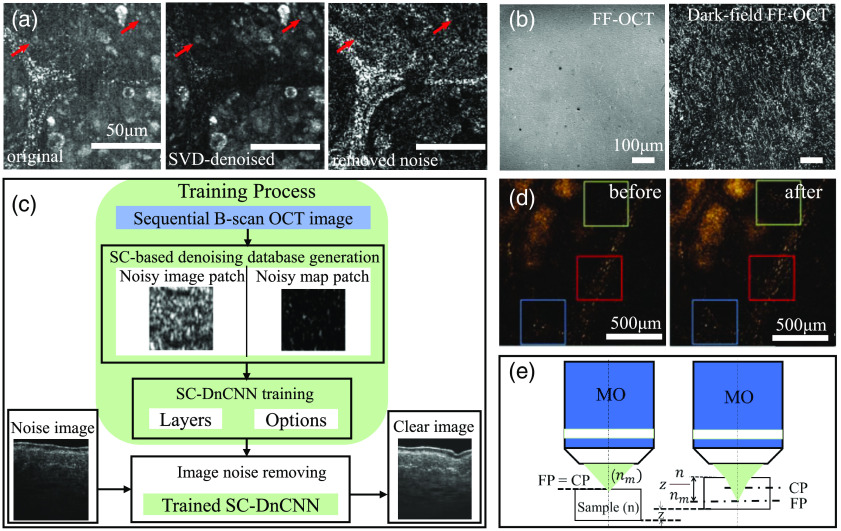
Image enhancement methods for FF-OCT. (a) The method based on SVD can effectively filter the motion artifacts in collagen fibers, rendering a clearer display of cells, pointed by the red arrow in the figure; (b) FF-OCT image and dark-field FF-OCT image recorded at the interface of cover glass and sheep brain tissue slice. Adapted from Refs. 49 and 56. (c) The process of deep learning network used to remove noise signals in FF-OCT images. Adapted from Ref. 57. (d) Comparison of FF-OCT images of a *Ficus* leaf before and after sample self-induced aberration was corrected when imaging at a depth of 75  μm. Adapted from Ref. 58. (e) The difference in refractive index between the microscope objective lens and air causes the coherence plane and the focal plane to separate. Adapted from Ref. 59.

Phase shifting and sample dithering during FF-OCT imaging may render motion artifacts and confuse image signals and noise, thereby impairing signal acquisition accuracy.^53^^,^^54^ The instantaneous phase shift without mechanical displacement mentioned in Sec. 3.1 is an approach to enhance the speed and accuracy of phase shifting as well as to reduce motion artifacts.^55^ And methods with two frames from the two outputs of the interferometer, *en face* tomographic images are calculated as a combination of two phase-opposed interferometric images acquired simultaneously by two CCD cameras placed at both outputs of the interferometer, with a spatial resolution of 0.8  μm×1.6  μm (axial × transverse). This new FF-OCT system, which uses instantaneous phase shifting with nonpolarizing optics and pulsed illumination, can suppress most of the artifacts that arise due to sample motion.^53^ Motion artifacts can also be reduced via image processing; as shown in Fig. 5(a). The method based on singular value decomposition (SVD) can effectively filter the motion artifacts in collagen fibers, rendering a clearer display of cells.^56^^,^^60^^,^^61^ When imaging biological tissue, an interface would occur between cover glass and tissues. If the refractive indexes of the two materials do not match, a strong “specular reflection” would occur, which can severely interfere with the recognition of backscattered signals of biological tissue. The dark-field FF-OCT can block specular reflections by the help of an opaque disk in the pupil-conjugated plane; the reference mirror is replaced by a blazed grating, which eliminates a walk-off between the sample and the reference beams on a camera that otherwise limits the imaging FOV, which suppresses specular reflection by at least two orders of magnitude.^49^^,^^62^ Therefore, high-contrast tomography can be captured at the cover glass–tissue interface. The results of dark-field detection are shown in Fig. 5(b), exhibiting a significantly increased contrast.

The coherence plane and the focus plane of the FF-OCT system are usually in the same plane. However, when imaging biological tissues with a dry objective, the difference between the refractive indexes of light in air ($n_{\mathrm{im}}$) and biological tissues (n) would separate the coherence plane and the focus plane, leading to defocus. As shown in Fig. 5(e), defocus would blur images reconstructed by FF-OCT. The differences in refractive index between the sample and the immersion medium of the microscope objectives may degrade the FFOCM image quality because of focus defect and optical dispersion mismatch. Dubois^59^ studied the effects of focusing defects and dispersion mismatch in full-field optical coherence microscopy. Performing water immersion or oil immersion objectives can ensure that $n_{\mathrm{im}}$ and n are close, for example, water immersion objectives can be used to mitigate defocus for imaging macrophages in Petri dishes.^19^^,^^41^ The multiscale aberration structure of biological tissues would diminish the imaging resolution of different depths. The adaptive optics (AO) system and OCT can be combined to correct the inherent aberration of biological tissues, in which AO devices can compensate for the signal loss caused by tissue wavefront distortion as well as improve the image clarity. More structure information can be observed due to AO correction, as the image shown in Fig. 5(d).^58^^,^^63^^–^^65^

Inherent speckle noise exists in FF-OCT, which would generate a grainy pattern and impair image quality. When it cannot be eliminated by hardware, certain image-processing algorithms can be adopted to reduce speckle noise, such as Gaussian filtering, median filtering, deep learning denoising, and other algorithms.^66^^,^^67^ In recent years, deep learning algorithms have achieved satisfying results in the noise reduction of FF-OCT images and paved a new way to improve FF-OCT image quality. The fundamental approach for noise elimination based on deep learning is to distinguish noise from signals by combining spatial mixing and noise spectrum prediction technology.^57^ As shown in Fig. 5(c), a neural network of two-phase image-spectrum conversion is adopted to reduce the noise of the FF-OCT image. By averaging adjacent B-scan images, speckles were reduced and the denoise database was obtained. A denoising convolution neural network based on the spatial-compounding (SC-DnCNN) was trained for noise recognition in the denoise database. After training, SC-DnCNN is used to eliminate the speckle noise of FF-OCT and output clean images.

### 3.3 Improving Imaging Throughput (FOV and Depth)

**Fig. 6:**
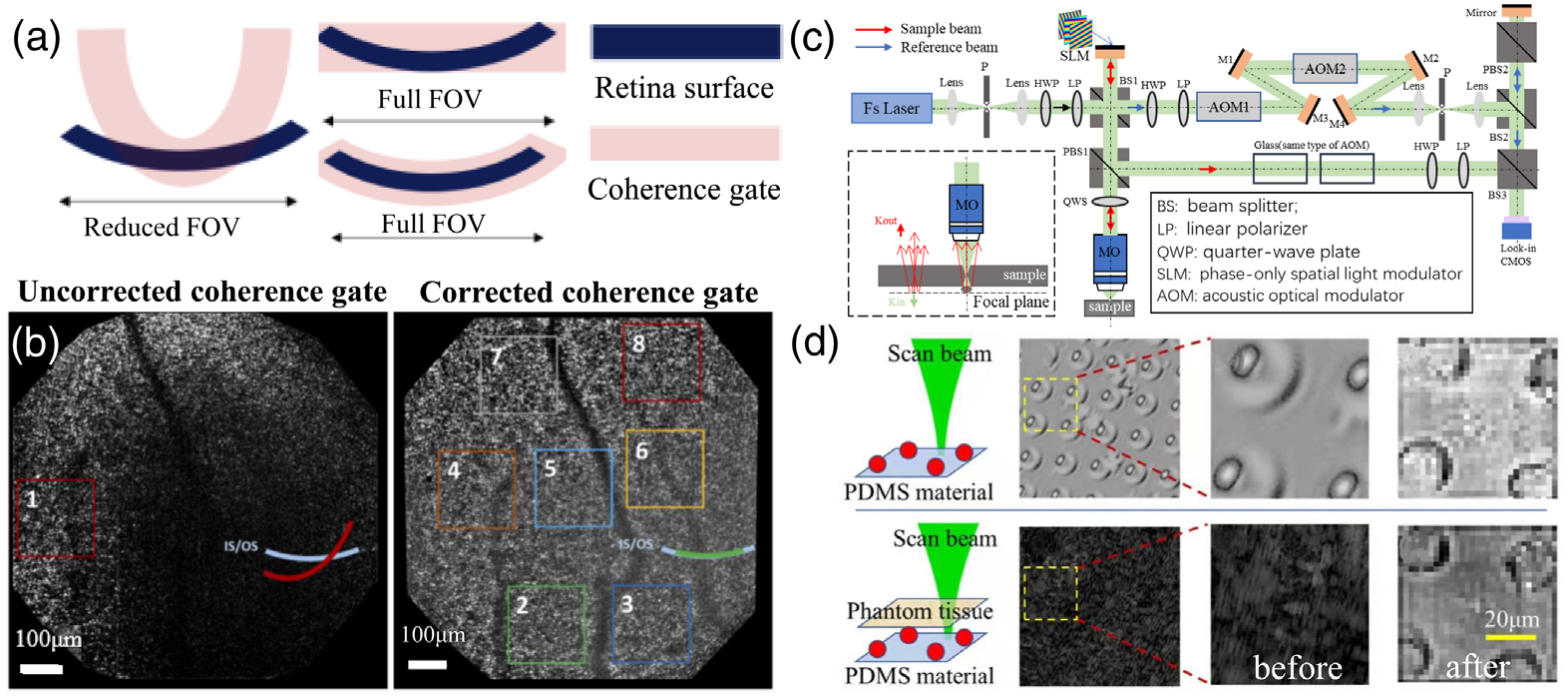
(a) Schematic showing different degrees of curvature for the coherence gate and their consequence on the FOV of the FF-OCT image, the left picture shows the coherence gate curvature does not perfectly match the retinal curvature, the middle picture shows the coherence gate is reshaped, although the curvature does not completely match the retinal curvature, a full FOV is still generated, as the coherence gate has a given thickness defined by the axial resolution. Adapted from Ref. 72. (b) The retina FF-OCT images before and after correcting for the coherence gate curvature. Adapted from Ref. 72. (c) FF-OCT imaging system based on coherent reflection matrix. Adapted from Ref. 74. (d) Samples with high scattering coefficient to verify deepening effect of imaging depth. Adapted from Ref. 74.

Large imaging range and high spatial resolution are the two goals expected to be simultaneously achieved for biomedical imaging, but mutual restraint exists between these two goals. To expand the imaging FOV of the system on the premise of ensuring a high spatial resolution of FF-OCT, a scanning mechanism can be added to the detection unit of the system for transverse stitching of tomograms. Alternatively, a multiconjugate AO structure can also be used to obtain a wider imaging FOV.^63^^,^^65^^,^^68^^–^^70^ The disadvantages of these approaches are as follows: transverse stitching of multiple elongates the imaging time, and the accuracy of image stitching is easily influenced by motion artifacts. The multiconjugate AO device increases the system complexity and shrinks the actual effective imaging field of a single AO device. Currently, coherent shaping is adopted to expand the effective imaging FOV of the FF-OCT system: the eye serving as the objectives breaks the symmetry of the Linnik interferometer in the FF-OCT system during retinal imaging, forming a curved coherent gate. Therefore, the effective imaging FOV of the FF-OCT system becomes smaller.^71^^–^^73^ The reference arm is equipped with an electric displacement platform to adjust the position of the coherent gate, and the sample arm is equipped with an optical window element to correct the geometry of the coherent gate. As shown in Fig. 6(a), the geometry of the coherent gate matches the curvature of the imaging sample, thereby expanding the effective imaging FOV. In retinal imaging, the effective imaging FOV after coherent gate shaping is three to four times larger than that before shaping [Fig. 6(b)].

Owing to the existence of aberration and multiple scattering, the imaging depth of FF-OCT for biological tissues is mostly limited to ∼1  mm, whereas the optimal imaging depth is limited to a few hundred microns. There are currently two approaches to increase the imaging depth of the FF-OCT in the scattering medium as much as possible. One approach utilizes optical clearing or other hardware, such as a light source with appropriate central wavelength based on the scattering properties of the sample or optical devices to reduce the imaging aberration. Another approach adopts a computational matrix, such as ballistic wave identification combining matrix calculation and iterative time reversal, which theoretically and experimentally confirmed that the imaging depth can be increased by at least two times.^75^ Chen’s group at University of California, Irvine (UCI) proposed a calculation method based on coherent reflection matrix measurement, which confirmed that the imaging depth can be extended. The reflection matrix is obtained by point-by-point scanning and phase shift, then SVD is conducted to retrieve the single backscattered light.^74^ Combining the reflection matrix measurement and wide-field heterodyne detection, satisfactory tomography of the microbeads through highly scattering medium is obtained with a doubled imaging depth; the system principle and imaging results are shown in Figs. 6(c) and 6(d).

### 3.4 Functional Imaging Expansion

Like OCT with spot scanning, FF-OCT can also develop multimodal composite imaging, which can be combined with one or more technologies such as dynamic imaging,^30^ fluorescence microscopic imaging,^24^^,^^25^ elastography,^76^ polarization-sensitive,^77^ and photothermal imaging^78^ and hyperspectral imaging.^79^ Therefore, multimodal images of one sample can be collected at the same position, which can not only provide structure information with subcellular-level resolution but also render data regarding tissue function.^23^^,^^25^^,^^31^

**Fig. 7:**
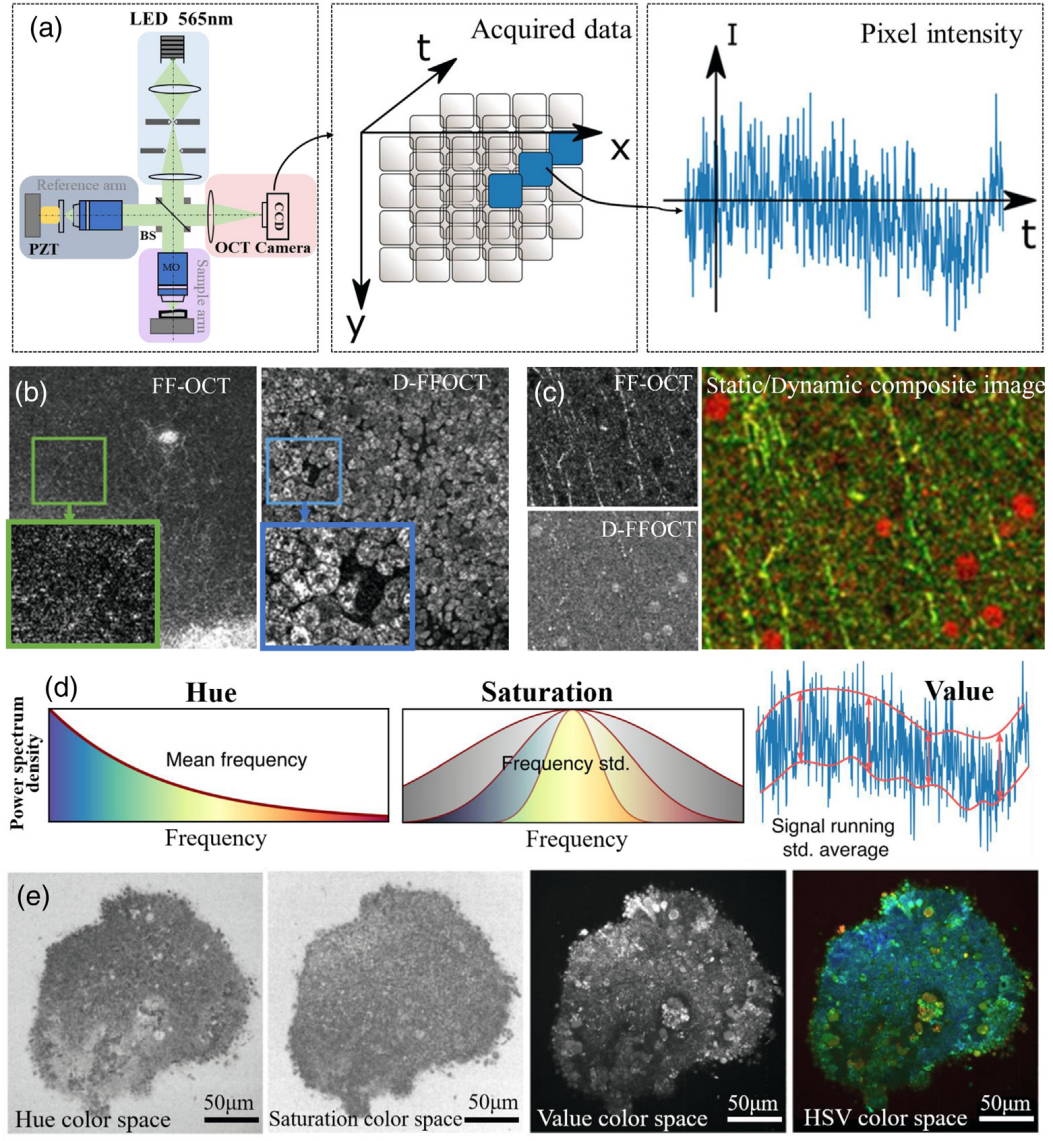
(a) Signal acquisition process of dynamic FF-OCT. Adapted from Ref. 27. (b) Static and dynamic FF-OCT images and partial enlarged images of rat liver. Adapted from Ref. 30. (c) Static/dynamic composite FF-OCT images of mouse brain cortex. Adapted from Ref. 30. (d) Dynamic images are computed in the HSV color space; hue is computed with the mean frequency, from blue (low temporal frequencies) to red (high temporal frequencies); saturation is computed as the inverse of the frequency bandwidth. The value is computed as the running STD. Adapted from Ref. 27. (e) Computation of the mean frequency (hue); frequency bandwidth (saturation); dynamic (value) grayscale image; dynamic FF-OCT of retinal organoid in the HSV color space. Adapted from Ref. 27.

As an extension of FF-OCT, dynamic functional imaging detects intracellular motility by analyzing the signal fluctuation. It provides a method of label-free cell imaging, which is a mostly a nondestructive detection method for tissue or cell dynamics compared with FM. As shown in Fig. 7(a) shows the signal acquisition process of dynamic FF-OCT, where the camera collects the interference images of the sample in temporal sequence and obtains a 3D data matrix. Temporal signal on each pixel is collected, and it is converted from the time domain to the frequency domain for power spectrum analysis. Dynamic FF-OCT uses cell motility as the endogenous contrast agent and exploits metabolically driven dynamic scattering changes of cellular structures to enhance contrast.^30^ Dynamic FF-OCT images serve as a useful supplement to static FF-OCT images, as static FF-OCT has high imaging contrast for static tissues such as fibers, blood vessels, and collagen, while dynamic FF-OCT is sensitive to cell membrane floating and cell surface reconstruction. Therefore, the superposition of static and dynamic FF-OCT images can enhance the overall visibility of tissue morphology. Due to the motion discrepancy between cytoplasm and connective tissue, dynamic FF-OCT images can clearly display the cell boundaries and demonstrate the hidden cells in static FF-OCT with high contrast. As shown in Fig. 7(b), the dynamic image of liver tissue exhibited clearer cell structures and boundaries than those in static images. The superposition of dynamic and static FF-OCT images of mouse brain tissue are shown in Fig. 7(c), which has significantly enriched the information capacity and contrast of the images.^30^

Dynamic FF-OCT imaging is calculated based on the standard deviation (STD) to obtain the interference signal fluctuation over time.^30^ The time-lapse sequence signal of the intensity $I(x,y,t_{i})$ is acquired and processed using Eq. (13):

$$D_{\mathrm{STD}}(x,y)=\sqrt{\frac{1}{N}\overset{N}{\underset{i=1}{\sum }}{\left(I(x,y,t_{i})-\overset{¯}{I}(x,y)\right)}^{2}},$$ (13)

where $I(x,y,t_{i})$ is the signal intensity and $\overset{¯}{I}(x,y)$ is the average of all signals in time dimension. To achieve better results of dynamic images, the time-lapse sequence signal $I(x,y,t_{i})$ is divided into multiple subdatasets; their STDs are first calculated separately, then several values are averaged. If the acquisition time interval between successive subdatasets is short, faster changes of movement can be captured while slower changes would be filtered; if the acquisition time interval is long, slower changes of movement can be captured while faster changes would be filtered.

To further enhance the display of dynamic images, the signal obtained by the STD method is analyzed by power spectrum to generate “frequency-power spectrum” data. The analysis results of STD power spectrum are displayed in different color spaces, reflecting cell motility. For instance, the cell dynamics can be divided into three normalized frequency bands by RGB color space, in which R, G, and B represent low, medium, and high frequency cell movements, respectively.^80^^,^^81^ Alternatively, the hue–saturation–value (HSV) color space can also be used, and the image brightness reflects the amplitude of cell movement while the image color is related to the cell motion speed.^27^^,^^29^ The HSV color display approach and the results of dynamic FF-OCT imaging of retinal organoids are demonstrated in Figs. 7(d) and 7(e). Colored dynamic images can demonstrate the overall activity distribution and local abnormal activity distribution of the sample.

In addition, dynamic imaging signals can also be analyzed using autocorrelation (AUTO),^82^ logarithmic intensity variance (LIV),^82^^–^^85^ and other methods. The specific calculation principles are provided in Eq. (14) and Eq. (15):

$$D_{\mathrm{AUCO}}(x,y)=\sqrt{(\frac{1}{N-1}\overset{N-1}{\underset{i=1}{\sum }}I(x,y,t_{i})I(x,y,t_{i+1}))-\overset{¯}{I}{\left(x,y\right)}^{2}},$$ (14)

$$D_{\mathrm{LIV}}(x,y)=\frac{1}{N}\overset{N-1}{\underset{i=0}{\sum }}{\left(10\;\log\;I(x,y,t_{i})-10\;\log\;\overset{¯}{I}(x,y)\right)}^{2}.$$ (15)

**Fig. 8:**
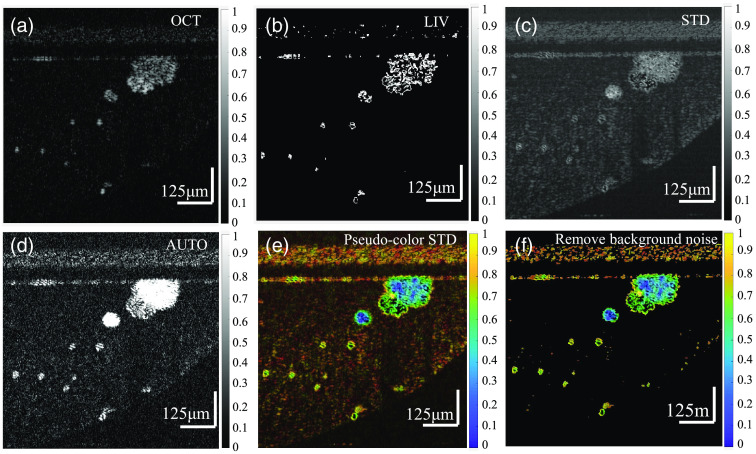
Cancer organoid imaging using different dynamic OCT method. (a) SD-OCT image of colorectal cancer organoids which are cultured in Matrigel. (b) Dynamic OCT image with LIV method. (c) Dynamic OCT image with STD method. (d) Dynamic OCT image with AUTO method. (d) Dynamic images in the HSV color space with STD method (256 fps SD-OCT images is used for calculation), where the orange-red background in the picture is due to the rapid movement of the inherent speckle noise of SD-OCT. (f) Dynamic images in the HSV color space with STD method with background noise removed using a mask. Scale bar: 125  μm.

By comparing the imaging results of various methods with data from the same sample, a more detailed intuitive understanding can be developed for each method. A data set of the colorectal cancer organoid (Organoid-O, Regenovo Ltd., Hangzhou, China) were acquired by exploiting the self-developed OCT equipment, and three dynamic imaging methods were adopted to process the same OCT data. The results are shown in Fig. 8. For the LIV method, the variance was calculated using the logarithm of intensity signals, and the static and dynamic components in the intensity signal were transformed into an additive form. Therefore, the static component can be easily subtracted by separating the moving tissue sample from the background [Fig. 8(b)]. For the STD method, the STD was directly calculated based on the intensity; thus, the images reflecting the motion frequency of samples were obtained. However, the speckle noise in the background cannot be well separated from organoids [Fig. 8(c)], which should be further processed, i.e., assigning color in the HSV channel according to the motion frequency [Fig. 8(e)] and eliminating the background noise [Fig. 8(f)]. The AUTO method makes the organoids with a low physical position in the image (far away from the focal position with low signal-to-noise ratio) obtain the signal intensity close to the sample at the focal plane and increases the overall signal-to-noise ratio of the image [Fig. 8(d)]. It must be highlighted that it is difficult to directly detect the dynamic discrepancy of different tissue layers using FF-OCT and D-FFOCT because only the *en face* tomography is obtained. Dynamic OCT imaging can directly visualize depth-related cell changes and structural maturity with time-spectroscopic analysis of cross-sectional tomography, which is particularly effective for the physiological and pathological analysis of epithelial tissue.^80^^,^^81^

### 3.5 Combination of FF-OCT Images and Deep Learning

Intelligent recognition of the important features or regions in FF-OCT images as well as the pathological or physiological information contained in images is a research focus in this field, which will bring revolutionary improvement to the clinical diagnostic application of FF-OCT.^86^

**Fig. 9:**
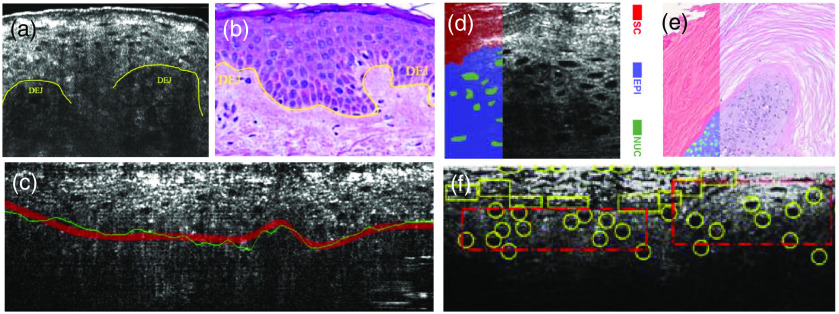
FF-OCT image processing using deep-learning techniques. (a) FF-OCT image of the DEJ of human skin. Adapted from Ref. 88. (b) Hematoxylin–eosin staining (H&E) image of DEJ of human skin. Adapted from Ref. 88. (c) Deep learning algorithm performs DEJ detection on FF-OCT images of human skin, where the red line is the DEJ manually marked by the doctor, and the green one is the automatic recognition result by the deep learning algorithm. Adapted from Ref. 88. (d) FF-OCT image of mouse squamous cell carcinoma skin, where the red region is the stratum corneum (SC), the purple region is the epidermis (EPI), and the green region is the nucleus (NUC). Adapted from Ref. 90. (e) H&E image of mouse squamous cell carcinoma skin; adapted from Ref. 90. (f) The result of deep learning tissue classification algorithm for squamous cell carcinoma detection in FFOCT images of mouse skin, where the yellow circles represent the identified cancerous cells. Adapted from Ref. 90.

In the latest research, deep learning has been exploited in the target recognition of FF-OCT images. For instance, U-net neural networks are used to identify red blood cells in skin microvessels from FF-OCT images with a subcellular-level resolution;^87^ multidirectional CNN is applied for predicting the possible pixels of epidermis in 3D data to enhance the accuracy of distinguishing dermal–epidermal junction (DEJ);^88^ the OCT image data of enhanced capillary network generated by automatic simulation algorithm are combined with the U-net neural network to achieve automatic segmentation of skin capillaries.^89^ The recognition of specific targets, particularly the recognition of structural features related to pathology, is crucial for the early diagnosis of severe diseases (e.g., cancer) and the in-depth understanding of tissue physiology and pathology. The combination of FF-OCT and deep learning can also enhance the accuracy of tissue segmentation [Fig. 9(c)]. A Pruned-ResNet-18 classifier based on the CNN network can be combined with FF-OCT images with subcellular-level resolution to detect skin squamous cell carcinoma, which has an accuracy of 80% in the early detection of this disease; the recognition results are shown in Fig. 9(f).^90^ Moreover, the automatic classification algorithm based on a support vector machine is exploited to distinguish the adipose tissue in the hollow structure of breast tissue; combined with the texture features from ultrahigh-resolution FF-OCT image, the invasive ductal carcinoma (IDC) tissue, and normal fibrous matrix can be further distinguished.^91^

The combination of high-resolution FF-OCT images and deep learning has especially revolutionized histopathological diagnosis, but it still has the shortcoming of manual marking, which is time- and labor-intensive, and the marking accuracy is influenced by precise identification of tissue characteristic margins. The colored images of cells and tissues with high resolution and contrast from dynamic FF-OCT can directly serve as characteristic marking images to train the deep learning network. Currently, based on feather engineering and the automatic diagnosis algorithm of CNN, some researchers performed the auxiliary diagnosis of cancer with an accuracy of nearly 100% using the high-resolution colored image of D-FFOCT at both small scale ($2\;{\mathrm{mm}}^{2}$) and macroscale ($2\;{\mathrm{cm}}^{2}$). This has verified the huge potential of combining D-FFOCT and deep-learning for fast, automatic, and accurate histopathological diagnosis.^92^

## 4 Applications of FF-OCT

Compared with FF-OCT, OCT has a larger imaging FOV and imaging depth and has been widely used in materials science, *in situ* process monitoring, and tissue engineering.^80^^–^^83^^,^^93^^–^^95^ But FF-OCT has isotropic micrometric resolution and has the ability of producing a histology-like image from a thick sample within a few milliseconds without physical slicing. Therefore, this technology is also known as optical biopsy. Benefiting from the advancement of the overall performance of the FF-OCT system, FF-OCT has appeared in many fields, such as biomedicine, materials science, security, and identification.

### 4.1 Biomedical Imaging Applications

**Fig. 10:**
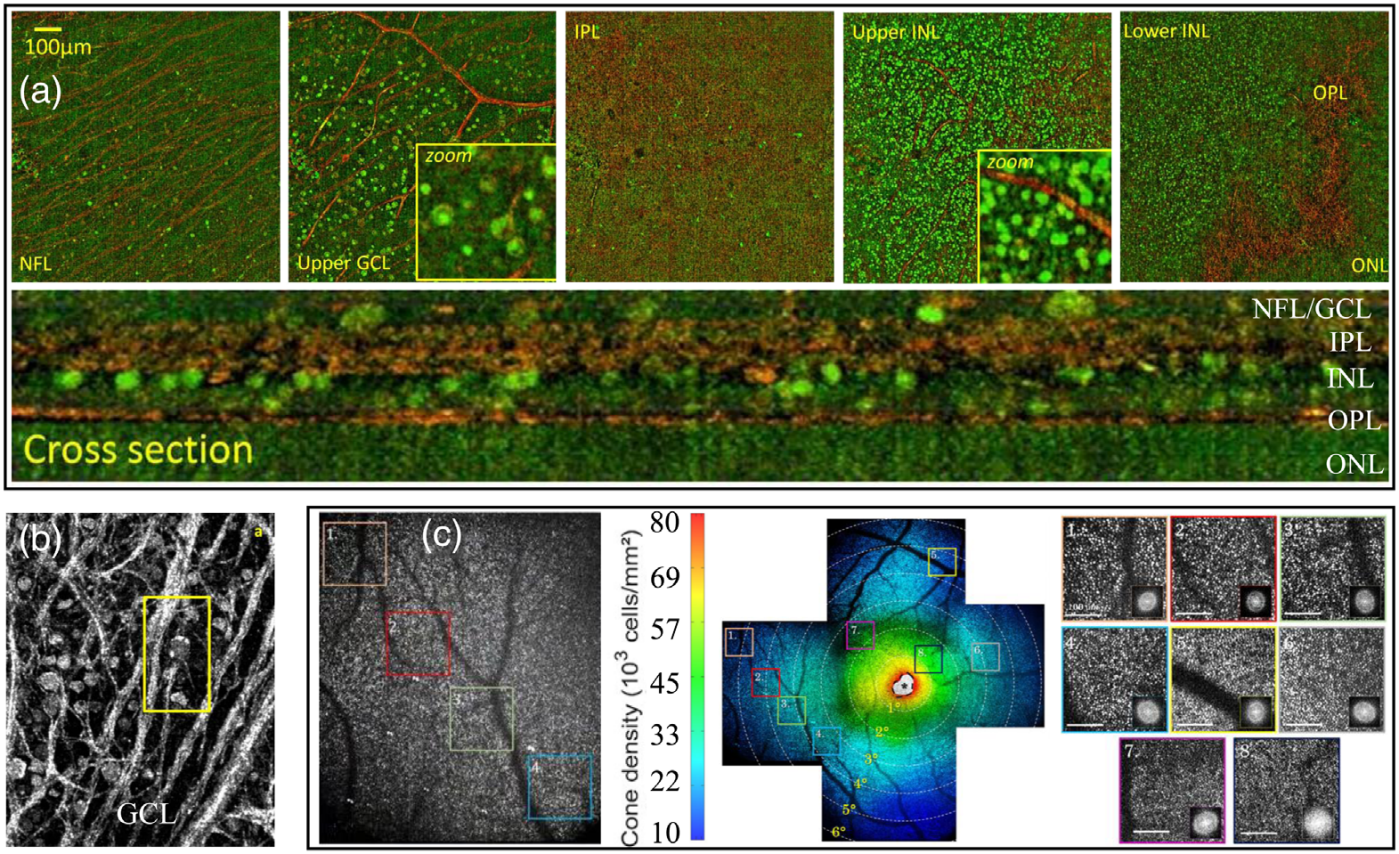
(a) Merged static (red) and dynamic (green) FF-OCT images on the macaque retina to identify stationary (fiber, blood vessel, collagen) and dynamic (cell) structures. Adapted from Ref. 28. (b) The FF-OCT image shows the details of the cell axons and cell bodies of the human retinal GCL, which can be used to check the details of the damage. Adapted from Ref. 96. (c) Combine AO and stitching methods to get the FF-OCT image of retina with a larger FOV. Adapted from Ref. 71.

FF-OCT can perform feature recognition from the microstructural level to cell level in ophthalmology, dermatology, oncology, and other fields. OCT has been most widely used in ophthalmology; compared with currently commercialized FD-OCT, FF-OCT can characterize cells and tissues as well as present pathological structure details.^31^^,^^54^^,^^65^^,^^71^^,^^72^^,^^96^^–^^100^ In the early stage of FF-OCT development, high-quality, high-resolution tomographic images can be obtained in some fields. For example, for retinal imaging, as early as 2004, some researchers used FF-OCT detected high-resolution retinal images and achieved the effect of distinguishing different structures.^15^ In addition, in other early applications, it also included tissue engineering scaffolds, etc.^101^ Even though high-quality imaging cannot be achieved due to hardware limits, FF-OCT opened a door for unique imaging performance, which made an important contribution to the development and application of FF-OCT. In recent years, imaging quality of FF-OCT has observed a significant improvement, Fig. 10(a) depicts the nerve fiber layer, ganglion cell layer (GCL), inner plexiform layer, outer plexiform layer (OPL), and outer nuclear layer (ONL) of macaque retina. Besides, due to its subcellular resolution, FF-OCT can detect the cellular characteristics of each layer within the retina such as the details of ganglion cell axons and cell bodies (yellow box) demonstrated in Figs. 10(a) and 10(b). Wide-field images are important for the early diagnosis of retinal diseases in clinical settings. Therefore, FF-OCT with a large imaging FOV, high resolution, and fast imaging speed has a prospective future in the field of ophthalmic detection. As shown in Fig. 10(c), the AO technology was adopted to obtain the retinal image of 5 deg×5 deg FOV, then five images were stitched into a 12 deg×12 deg FOV via a certain stitching method, in which the color map represents the calculated photoreceptor density. Both coherent gate shaping and AO technology can be used to expand the imaging field of FF-OCT during retinal detection. Moreover, optical phase modulation via movement of the eye itself to retrieve tomographic images of TD-FFOCT has also been achieved. The combination of this method with coherent gate shaping technology has the potential to facilitate the clinical application of FF-OCT in the ophthalmology field.^54^

**Fig. 11:**
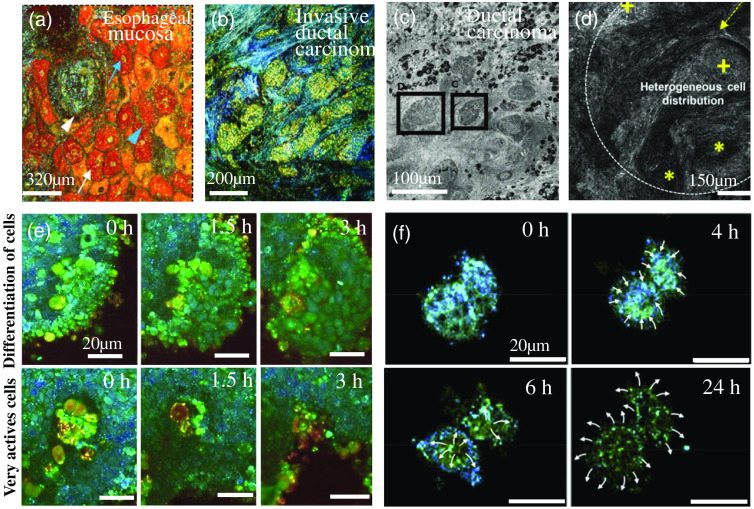
(a) D-FFOCT image of esophageal mucosa at a 10  μm depth, where the epithelial cell cytoplasm (white arrows) and nuclei (blue arrows), pericellular lining (blue arrowheads), lamina propria papillae (white arrowheads). Adapted from Ref. 109. (b) D-FFOCT image of invasive ductal carcinoma of breast tissue, in which nested cells with malignant features have local low intensity. Adapted from Ref. 102. (c) FF-OCT image of mouse skin squamous cell carcinoma where the cancerous tissue in the black box. Adapted from Ref. 103. (d) FF-OCT image of head and neck cancer, where the yellow dotted arrow points to the stromal reaction, the yellow cross mark points to the cancerous tissue, and the yellow asterisks points to the keratin pearls. Adapted from Ref. 104. (e) In the first line, D-FFOCT monitors the dynamic profile (the boundary between the two cells) changes during cell differentiation; the second line, the very active area in the organoid, is composed of cells that exhibit high dynamics, and may be undergoing apoptosis. Adapted from Ref. 27. (f) D-FFOCT monitors the activity and morphology of Hela cells over time. Adapted from Ref. 32.

Combining FF-OCT with other imaging technologies to perform multimodal image acquisition at the same position of the sample can significantly enrich imaging contrast and provide functional information besides structure. Therefore, FF-OCT performance in medical diagnosis can be greatly enhanced, exhibiting clinical potential in early cancer diagnosis and serving as guidance in tumor resection. As shown in Figs. 11(a)–11(d), the reported applications include, but are not limited to, the discrimination of benign and malignant tissues of skin cancer,^35^^,^^103^^,^^105^ breast invasive ductal cancer,^102^^,^^106^ head and neck cancer,^104^ as well as optical biopsy of intestinal tissues,^107^^,^^108^ detection of esophageal mucosa, liver, and gastric mucosa.^86^^,^^102^^,^^106^^,^^109^^–^^113^ Besides, FF-OCT incorporating artificial intelligence can benefit deep mining of the abundant image data, which can reduce the difficulty of human eye recognition, improve the accuracy of information recognition, and promote the efficiency of FF-OCT histological diagnosis.

FF-OCT can provide high-resolution structural and functional information of 2D or 3D cell cultures and 3D-printed cell scaffolds under a normal culture environment.^15^^,^^34^^,^^114^ Researchers have used dynamic FF-OCT to conduct long-term longitudinal comparative studies on organoid samples, including long-term observation of cell apoptosis, proliferation, or migration as well as observation of the dynamic contour changes of tissues during organoid cell differentiation [Fig. 11(e)]. Moreover, the dynamic and static dual-mode of FF-OCT detection has also been exploited to monitor the changes in cell viability in cancer cells (e.g., Hela cells) and complex culture systems [Fig. 11(f)]. The ability of FF-OCT to track the growth and development of cells *in vitro* can monitor the cell viability change in disease modeling or artificial tissue transplantation as well as reveal the mechanism of structural and functional reconstruction of 3D cultured tissues such as organoids and tumor microspheres, thereby facilitating research on the mechanism of human development and disease.^15^^,^^26^^,^^27^^,^^32^^,^^34^^,^^114^ The functional imaging extension of FF-OCT is also applied in spectroscopic FF-OCT, with different pseudocolors showing melanocytes in *ex vivo* *Xenopus laevis* tadpole embryos at high resolution.^115^^,^^116^ In addition, it also includes superimposing the fluorescence microscopic image of the mouse colon and the FF-OCT image, so more information can be displayed in one image;^117^ the images obtained at the same time at the center wavelength of 800 and 1200 nm are given different colors, respectively. Superimposed multimodal imaging was applied to detect rabbit trachea, which can increase the contrast of different tissue structures, significant improvement in imaging penetration depth was demonstrated at 1200 nm compared to 800 nm, and a color image representation was applied to fuse both images into a single image for better comparison of wavelength effects.^118^

### 4.2 Expansion of FF-OCT Applications

**Fig. 12:**
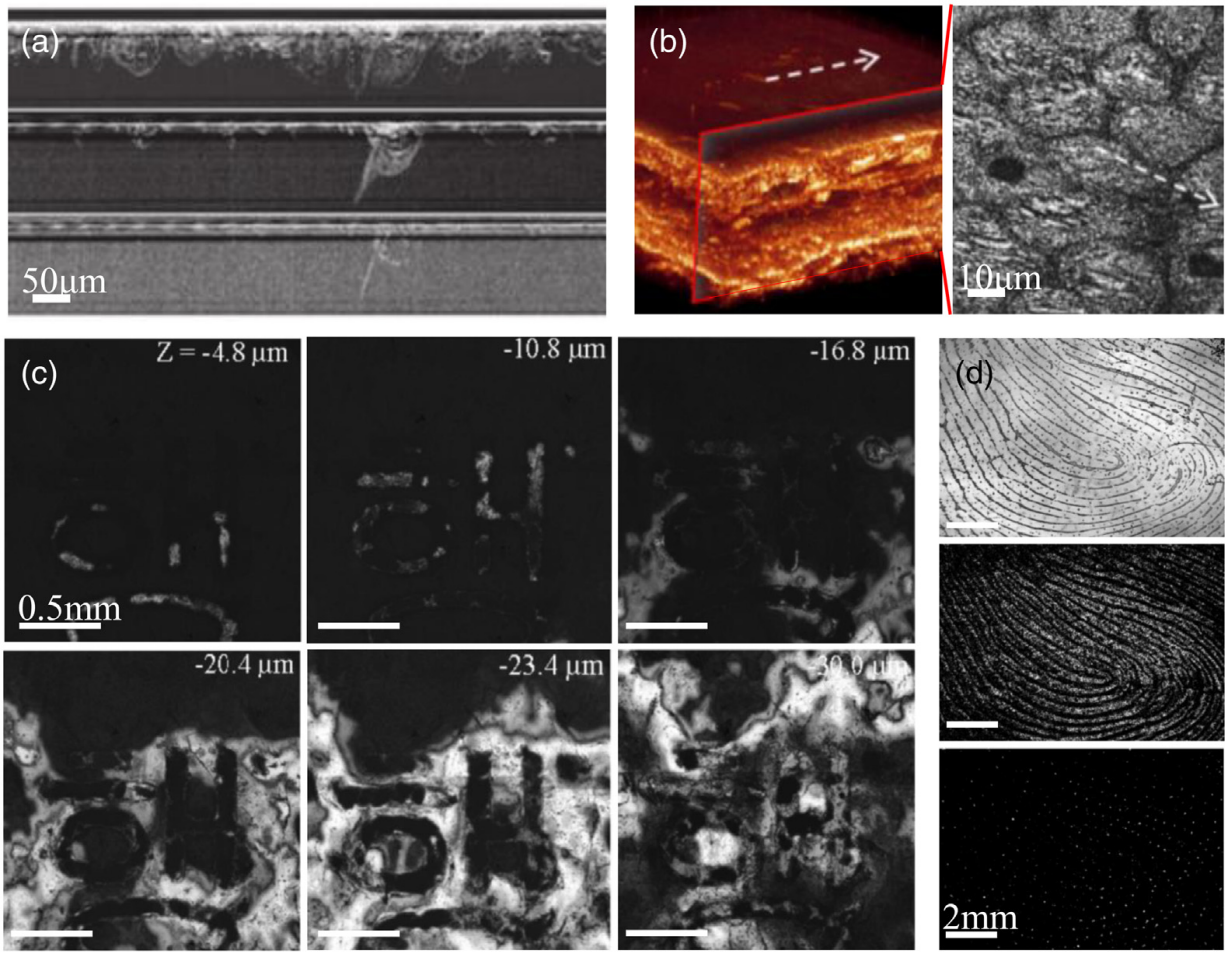
(a) Subsurface damage of optical glass using FF-OCT. Adapted from Ref. 122. (b) Human hair damage detection. Adapted from Ref. 123. (c) FF-OCT cross-sectional views of different depths of banknotes, used for authenticity identification. Adapted from Ref. 124. (d) FF-OCT fingerprint patterns at different depths, from top to bottom are external fingerprints (0  μm), internal fingerprints (300  μm), and sweat pores (200  μm). Adapted from Ref. 125.

Benefiting from the enhancement of the overall performance of FF-OCT system, its potential in scientific research and application of materials science, security, and identification has gradually been valued by researchers. FF-OCT can nondestructively acquire 2D tomography of a certain depth with high axial and transverse resolution.^119^^–^^121^ Therefore, this technology can detect the specific spatial location and detailed characteristics of material damage to analyze the degree and cause of the damage. For instance, FF-OCT can be used to assess the subsurface (dozens of microns below the surface) damage of optical glass [Fig. 12(a)] and observe the internal and external damage of hair [Fig. 12(b)].^122^^,^^123^

The unique internal characteristics of security objects can be adopted as new indicators of anticounterfeiting measures. Noninvasive detection of the internal details of banknotes by FF-OCT can be used to identify their authenticity [Fig. 12(c)].^124^ The internal fingerprint detection can function as identity recognition or a password [Fig. 12(d)], which is more secure than the surface fingerprint. Modern forgery is highly specialized, and the appearance of imitations may be mistaken as genuine.^124^^–^^127^ Therefore, FF-OCT is attracting increased attention as an effective tool for authenticity identification of certificates, paintings, and porcelain, exhibiting a broad range of application prospects in anticounterfeiting science.

## 5 Summary and Discussion

This work explicitly introduced the working principle and performance parameters of the FF-OCT system and summarized the latest advances and applications of FF-OCT performance optimization. Relevant technologies for increasing the imaging speed (acquisition, phase shift, and reconstruction), imaging quality (optimization of contrast and signal-to-noise ratio), and imaging throughput (extension of imaging FOV and depth), as well as adding complementary contrast to structural images and information mining have also been detailed in this work.

Although FF-OCT has undergone significant progress, the following aspects require in-depth investigation, which could become the focus of innovative research in this field:

(1) Combining optical field control technology and computational optics to extend the actual detection depth of FF-OCT. The transverse and axial resolution of FF-OCT can reach submicron levels, but the effective imaging depth is limited to 1 mm due to multiple scattering and aberration of the sample itself. The FF-OCT imaging method, which is based on measuring of the time-gated reflection matrix of a modulated optical field and matrix SVD theory, can eliminate a majority of the multiple scattering and effectively extend the imaging penetration depth.

(2) Enhancing the information capacity and information extraction efficiency of FF-OTC images by mathematical methods or signal processing. Multimodal OCT is a conventional method to achieve structure–function composite imaging; however, owing to the difference in acquisition methods, the FF-OCT optical system must be redesigned when incorporating laser confocal, two-photon, and other imaging technologies. If the information in the existing spectral interference signals can be mined from the perspective of signal processing, the contrast of the original image can be increased and new functional information can be added. For instance, dynamic FF-OCT images can more clearly display cell boundaries and cell activity states with high contrast. Further, the combination of deep learning and super-resolution FF-OCT images with high information capacity can efficiently mine the valuable information of images, thereby exhibiting remarkable application prospects in early cancer diagnosis and histological diagnosis.

(3) Expanding the applications of FF-OCT. FF-OCT imaging extends the detection depth of ultrahigh-resolution imaging and can be utilized in nondestructive continuous detection for organoids, cell spheres, and artificial tissues in subcellular level. Moreover, it provides a new tool for in-depth investigation of the growth and development regularity of tissues and organs *in vitro*. In addition, FF-OCT can detect the characteristics of samples at a specific depth, thereby functioning as a rapid detection approach for flaw detection of material subsurfaces, safe-guarding, anticounterfeiting, art identification, and other fields.
